# Supplementary material for: Antioxidant and Antimicrobial Activity of Algal and Cyanobacterial Extracts: An In Vitro Study
Source: Antioxidants (Basel). 2022 May 19;11(5):992. doi: 10.3390/antiox11050992 (PMC9137800; doi:10.3390/antiox11050992)
Supplement: Supplementary file 1 [file antioxidants-11-00992-s001.zip › antioxidants-1715042-supplementary.pdf]

Supplementary Materials

Tables

Table S1. Percentage of inhibition (PI%) of radical scavenging activity of three different concentrations (100%; 75%; 50%) of four algal and cyanobacterial extracts tested *(Arthrospira platensis; Ascophyllum nodosum; Chlorella vulgaris; Lithothamnium calcareum* and *Schizochytrium* spp.).

	Percentages of inhibition (PI%)		
**Concentrations of algal extracts**	**100%**	**75%**	**50%**
Arthrospira platensis	19.66 ± 1.76 [a]	16.91 ± 0.17 [b]	12.60 ± 0.40 [c]
Ascophyllum nodosum	57.75 ± 1.44 [a]	49.49 ± 0.59 [b]	43.30 ± 1.69 [c]
Chlorella vulgaris	9.80 ± 1.23 [a]	6.27 ± 0.24 [b]	5.51 ± 0.95 [c]
Lithothamnium calcareum	2.97 ± 0.14 [a]	2.43 ± 0.10 [b]	1.84 ± 0.08 [c]
Schizochytrium spp.	18.27 ± 0.55 [a]	15.23 ± 2.73 [b]	10.86 ± 0.46 [c]
p-value	0.0001	0.0001	0.0001

Results are expressed as percentage of inhibition (PI%). Data are shown as means and standard deviations.
[a,b] Means (n=3) with different superscripts are significantly different (Treatment $p < 0.0001$).

Table S2. Algae and cyanobacterium combination and their synergic effect. The table show all the algae and cyanobacteria combination tested, and their respective antioxidant effect either as the sum of the effect obtained by each individual alga at 50% concentration, or as a synergistic effect.

	Percentages of inhibition (PI%)		
	**Sum of single effect at concentration of 50%**	**Combined algal extract**	***p* value**
Arthrospira platensis + Lithotamnium calcareum	14.45 ± 0.40[a]	9.37 ± 0.71[b]	0.0001
Chlorella vulgaris + Lithothamnium calcareum	6.27 ± 0.24[a]	9.59 ± 1.10[b]	0.0001
Chlorella vulgaris + Schizochytrium spp.	15.29 ± 0.61[a]	12.07 ± 0.74[b]	0.0001
Lithotamnium calcareum + Schizochytrium spp.	12.69 ± 0.50	12.11 ± 0.87	0.1067
Arthrospira platensis + Chlorella vulgaris	17.05 ± 0.39[a]	13.01 ± 0.28[b]	0.0001
Ascophyllum nodosum + Chlorella vulgaris	47.70 ± 1.79[a]	13.11 ± 0.52[b]	0.0001
Arthrospira platensis + Schizochytrium spp.	23.47 ± 0.32[a]	16.98 ± 0.48[b]	0.0001
Arthrospira platensis + Ascophyllum nodosum	55.89 ± 1.93[a]	19.67 ± 2.97[b]	0.0001
Ascophyllum nodosum + Schizochytrium spp.	54.12 ± 1.79[a]	38.74 ± 0.55[b]	0.0001
Ascophyllum nodosum + Lithothamnium calcareum	45.10 ± 1.04[a]	48.84 ± 1.37[b]	0.0001

Results are expressed as percentage of inhibition (PI%). Data are shown as means and standard deviations. [a,b] Means (n=3) with different superscripts are significantly different (Treatment $p < 0.0001$).

Figures

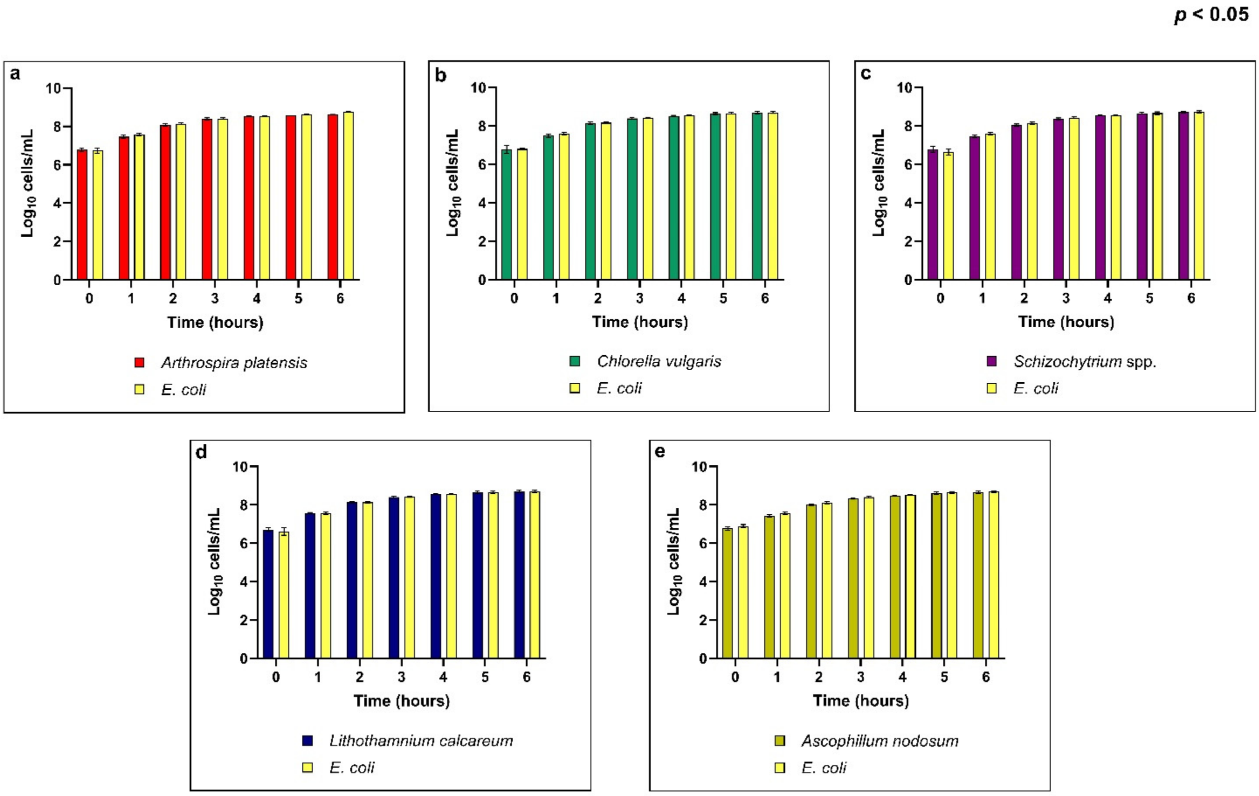

Figure S1: Growth inhibition of algal and cyanobacterial extracts diluted at 1:32 against *E. coli*. (a) Growth inhibition of *Arthrospira platensis*. (b) Growth inhibition of *Chlorella vulgaris*. (c) Growth inhibition of *Schizochytrium spp*.(d) Growth inhibition of *Lithothamnium calcareum* (e) Growth inhibition of *Ascophyllum nodosum*. Data are shown as means and standard deviations (Treatment $p < 0.05$).

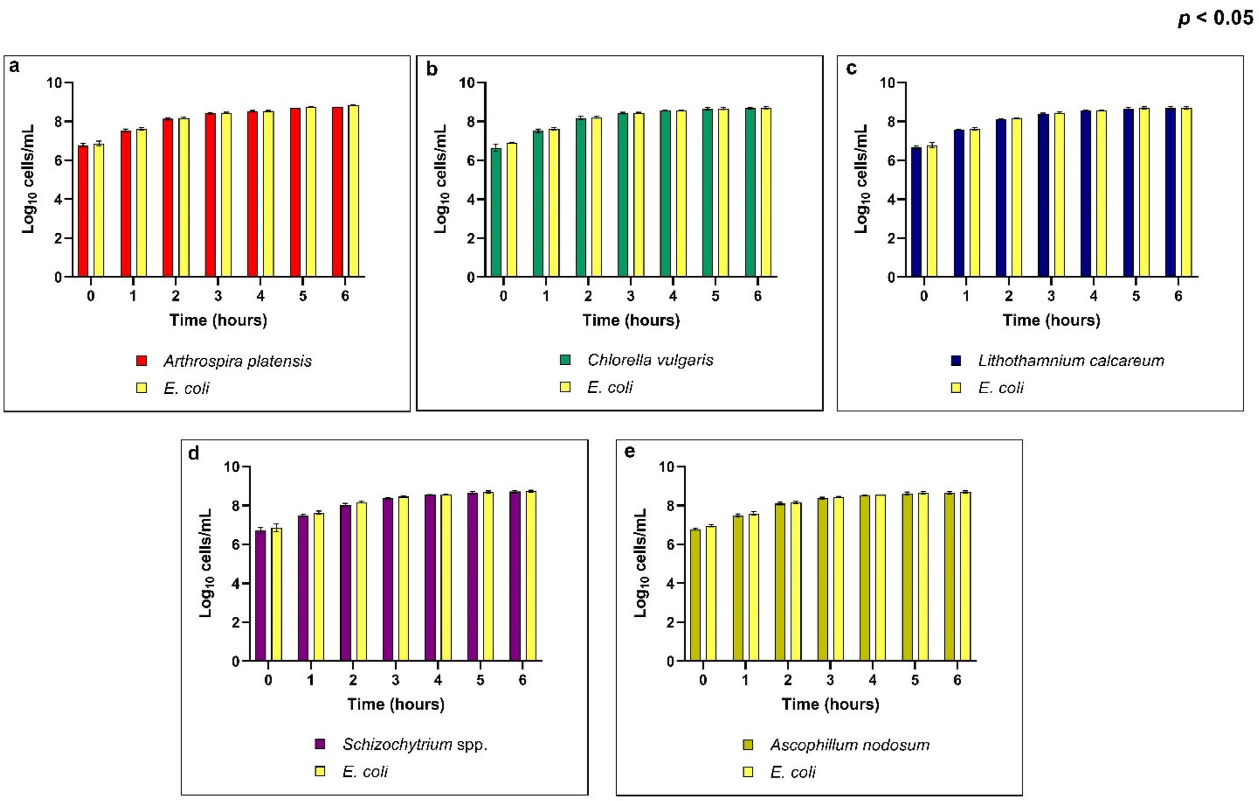

Figure S2: Growth inhibition of algal and cyanobacterial extracts diluted at 1:64 against *E. coli*. (a) Growth inhibition of *Arthrospira platensis*. (b) Growth inhibition of *Chlorella vulgaris*. (c) Growth inhibition of *Schizochytrium* spp. (d) Growth inhibition of *Lithothamnium calcareum* (e) Growth inhibition of *Ascophyllum nodosum*. Data are shown as means and standard deviations (Treatment $p < 0.05$).

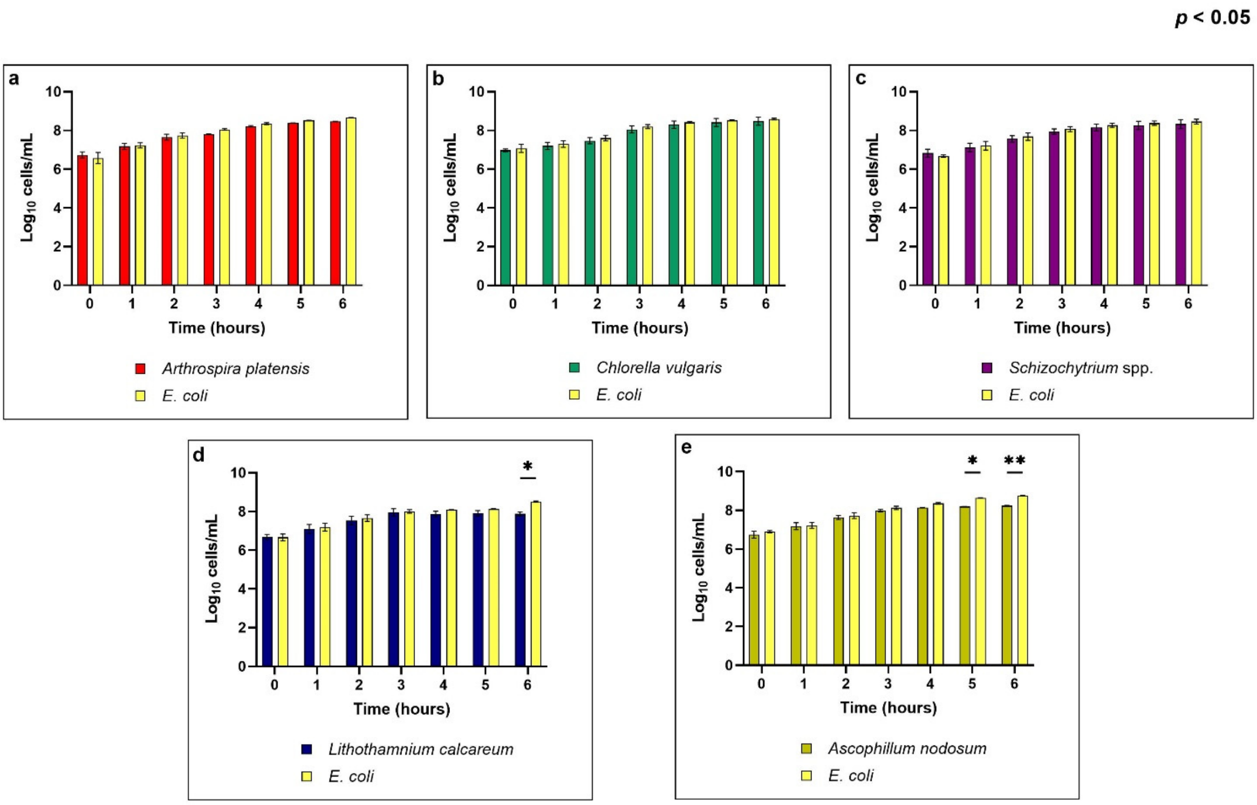

Figure S3: Growth inhibition of algal and cyanobacterial extracts diluted at 1:8 against *E. coli*. (a) Growth inhibition of *Arthrospira platensis*. (b) Growth inhibition of *Chlorella vulgaris*. (c) Growth inhibition of *Schizochytrium* spp. (d) Growth inhibition of *Lithothamnium calcareum* (e) Growth inhibition of *Ascophyllum nodosum*. Data are shown as means and standard deviations. * Asterisks means (n=3) with different superscripts are significantly different (Treatment $p < 0.05$)

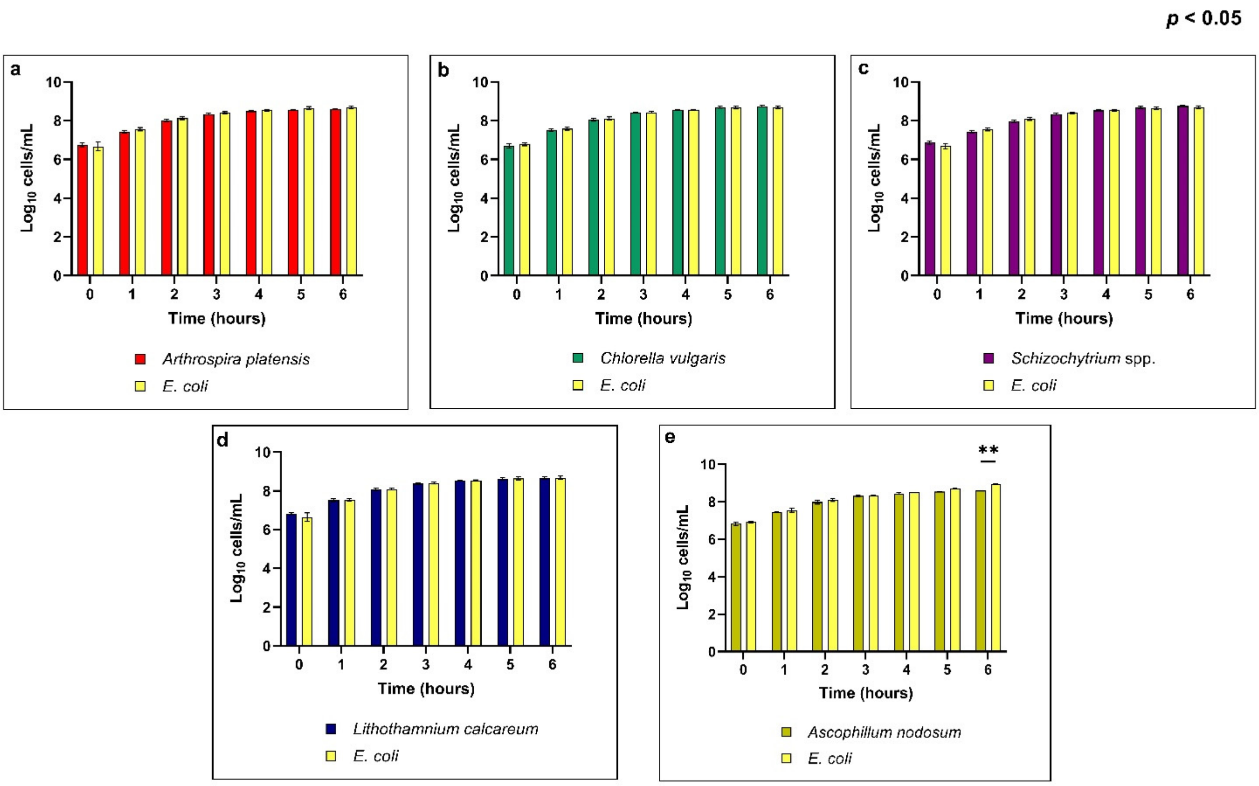

Figure S4: Growth inhibition of algal and cyanobacterial extracts diluted at 1:16 against *E. coli*. (a) Growth inhibition of *Arthrospira platensis*. (b) Growth inhibition of *Chlorella vulgaris*. (c) Growth inhibition of *Schizochytrium* spp. (d) Growth inhibition of *Lithothamnium calcareum* (e) Growth inhibition of *Ascophyllum nodosum*. Data are shown as means and standard deviations. * Asterisks means (n=3) with different superscripts are significantly different (Treatment $p < 0.05$).

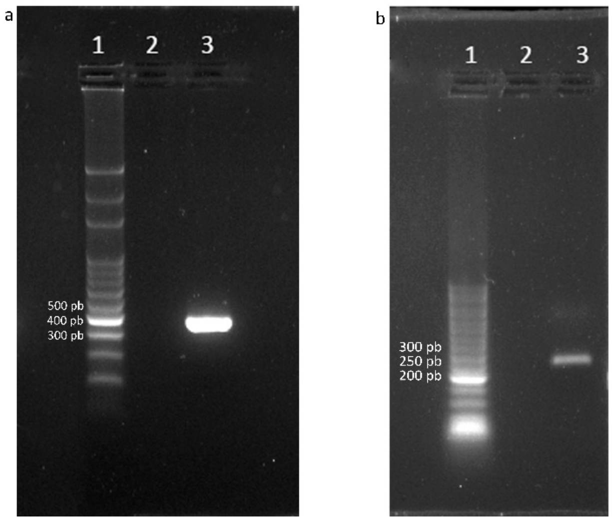

Figure S5. (a) Agarose gel (1.5%) one-dimensional electrophoresis of PCR products for the detection of FedF gene from genomic DNA of O138 *Escherichia coli* strain. Lane 1: marker 100 bp; lane 2: negative sample for the detection of FedF; lane 3: positive sample for the detection of FedF. (b) Agarose gel (1.5%) one-dimensional electrophoresis of PCR products for the detection of VT2eB gene from genomic DNA of O138 *Escherichia coli* strain. Lane 1: marker 50 bp; lane 2: negative sample for the detection of VT2eB; lane 3: positive sample for the detection of VT2eB.
